# Experience with the management of 2599 cases of congenital muscular torticollis and a multicenter epidemiological investigation in 17 hospitals in China

**DOI:** 10.1186/s12891-023-06983-w

**Published:** 2023-11-18

**Authors:** Zhenhui Zhao, Hansheng Deng, Yuanheng Li, Xinyu Wang, Gen Tang, Yueping Zeng, Hui Xu, Qisong Yang, Zhengyu Wu, Shicheng Li, Zhiwen Cui, Guoshuang Feng, Guibing Fu, Shengping Tang, Zhu Xiong, Xin Qiu, Futang Research Center of Pediatric Development(FRCPD), Jian Tian, Fei Song, Xin Xu, Mei Wu, Guosong Wang, Li Li, Hongjie Sun, Zhenqiang Da, WenJuan Wang, Qinghong He, Shaoqian Liu, Ling Dai, Waiguang Hu, Xiaoqin Wang, Jian Du, Chunxiang Wang, Yuanyi Qu, Daqiao Zhu, Jian Ding, Haibin Zhou, Jinchi Shi, Zhijun Pan, Lei Yang, Tingting Zhang, Jin Xu, Lianjun Ruan, Shu Mai, Fengmei Ma, Li Gao, Hongcheng Liu, Xirong Chen, Yuzheng Zhang, Jun Zhou, Chun Xiang Yan, Jian Fang

**Affiliations:** 1 Guangdong Province, Shenzhen Pediatrics Institute of Shantou University Medical College, Shenzhen Children’s Hospital, Shenzhen, P. R. China; 2 China Medical University, Shenyang, Liaoning Province P. R. China; 3 Department of Biomedical Sciences, University of Sassari, 07100 Sassari, Italy; 4 Orthopaedic Department, Sassari University Hospital, 07100 Sassari, Italy; 5 CAS Key Laboratory of Human-Machine Intelligence-Synergy Systems and the SIAT Branch, Shenzhen Institute of Artificial Intelligence and Robotics for Society, Shenzhen, Guangdong Province P. R. China; 6 The Guangdong-HongKong-Macau Joint Laboratory of Human-Machine Intelligence-Synergy Systems, Shenzhen Institutes of Advanced Technology (SIAT), Chinese Academy of Sciences, Shenzhen, Guangdong Province P. R. China; 7 Big Data Center, Beijing Children’s Hospital, Capital Medical University, National Center for Children’s Health, Beijing, China; 8 Hefei Center for Disease Control and Prevention, Hefei, P. R. China; 9 Hefei Cancer Hospital, Chinese Academy of Science, Hefei, P. R. China

**Keywords:** Congenital muscular torticollis, Management, Physiotherapy

## Abstract

**Background:**

Congenital muscular torticollis (CMT) is a common musculoskeletal disease affecting infants and young children. If CMT is not treated correctly and timely, it can lead to limited head and neck movements, head and neck deviation, and abnormal posture. In order to improve patients' symptoms and alleviate the negative impact of the disease on their lives, we are committed to exploring the treatment of CMT.

**Methods:**

The general clinical and ultrasonographic data of 2599 children with CMT who received standardized treatment at Shenzhen Children’s Hospital from 2004 to 2020 were retrospectively reviewed. According to given treatment, children with CMT were divided into the physiotherapy group, physiotherapy combined with glucocorticoid treatment group, and surgical treatment group. We divided children with CMT into local mass, uniform thickening, and atrophy according to ultrasound features. General clinical information, treatment, and ultrasound examination data in each group were compared. Additionally, electronic medical records of 2344 patients admitted due to CMT in 17 tertiary children’s hospitals of China’s Futang Research Center of Pediatric Development (FRCPD) from 2015 to 2019 were retrospectively analyzed. Data on sex, age, year of admission and discharge, and treatment costs during hospitalization were extracted from the first medical record pages according to the ICD codes. The data were assessed for normality using the Kolmogorov–Smirnov test. Depending on the data distribution, they were analyzed using parametric tests, such as the t-test, or non-parametric tests. Qualitative data are expressed as percentages (%) and analyzed using the chi-square or Fisher’s exact probability test, with α = 0.05 as the test level. *P* < 0.05 was considered to be indicative of a statistically significant difference.

**Results:**

Three types of CMT were defined based on sternocleidomastoid muscle ultrasound examination characteristics: local mass, uniform thickening, and atrophy. Age at first diagnosis was 69.21 ± 108.41 days in local mass type group, 216.85 ± 324.09 days in uniform thickening group, and 417.88 ± 739.05 days in atrophy- type group; while age at first physiotherapy use was 94.06 ± 206.49 days, 255.00 ± 430.62 days, 540.92 ± 1059.29 respectively. The children included in local mass type group have shown a high success rate of conservative treatment, with a rate of 7.5% of children underwent surgery. Age at first diagnosis was 112.44 ± 224.12 days in the physiotherapy group, 115.87 ± 144.86 days in the physiotherapy combined with glucocorticoid treatment subgroup, whereas the age at first physiotherapy use was 137.38 ± 312.11 and 196.91 ± 344.26 days respectively. In the observation period (2015–2019) the mean age at surgery for CMT in 17 tertiary children’s hospitals of the FRCPD was 50 months. Overall, 663 children with CMT were 1–2 years of age, accounting for the largest proportion (28.3%). Followed by 417 individuals (17.8%) were 7–14 years of age, indicating that there are still more children with CMT receiving surgical treatment later.

**Conclusions:**

Early diagnosis and treatment are essential to improve the conservative treatment success rate and achieve good prognosis in children with CMT. Our team’s concept for treating CMT is as follows: after diagnosing the children, we will adopt the standardized protocol of treatment, with physiotherapy combined with the injection of glucocorticoid drugs and SCM release surgery, when needed. This program has a high conservative treatment success rate and may facilitate the achievement of better prognosis and reduced teratogenicity rate.

## Introduction

Congenital muscular torticollis (CMT) is caused by fibrosis on one side of the sternocleidomastoid muscle (SCM) as well as skeletal muscle contracture, resulting in limited head and neck movements, head and neck deviation, and abnormal posture [1–3]. Without timely diagnosis and treatment, CMT can lead to facial asymmetry and spinal deformity [4–6]. The reported incidence of CMT varies from less than 1% to 3.92% worldwide [7–9]. While the CMT’s pathogenesis remains controversial, it may involve birth trauma, abnormal fetal position, infection, and SCM dysplasia [4, 10, 11].

Treatment of CMT, which includes conservative and surgical approaches, aims to relieve neck movement limitations, eliminate SCM mass, correct head and neck skewing, and prevent facial and cranial deformities [12]. Conservative treatment methods include simple observation, physiotherapy, drug injection therapy, home-based rehabilitation training, orthotic application, and traction [12–14]. On the other hand, standard surgical methods for treating CMT include unipolar and bipolar SCM release, partial or complete resection of the SCM, Z-shaped SCM lengthening, and laparoscopic treatment [15–17].

Nonetheless, the choice between treatment options and the timing of surgery for CMT in children remain controversial. Summarizing the treatment options for CMT will aid in promoting a standardized treatment for CMT. The present study aimed to retrospectively analyze the general clinical and ultrasonographic data of 2599 children with CMT who received standardized treatment protocol at our hospital from 2004 to 2020. Additionally, electronic medical records of 2344 patients admitted due to CMT in 17 tertiary children’s hospitals of China’s Futang Research Center of Pediatric Development (FRCPD) from 2015 to 2019 were retrospectively analyzed. According to given treatment, children with CMT were divided into the physiotherapy group, physiotherapy combined with glucocorticoid treatment group, and surgical treatment group. The baseline data, treatment, and ultrasound-related characteristics of the children in each group were compared to examine the clinical characteristics and prognoses of children with CMT across different groups. Furthermore, combined with our hospital's many years of experience in the management of CMT, a standardized treatment protocol for children with CMT has been constructed. After the patient is diagnosed, we will apply regular physiotherapy, with glucocorticoid injections and SCM release surgery added when necessary. This protocol improves the success rate of conservative treatment.

## Materials and methods

### Ethical review statement

This study was carried out in full compliance with the Declaration of Helsinki. This study has been approved by the Medical Ethics Committee of the Shenzhen Children’s Hospital (No. 202000302). All patients and their legal guardian/Parents voluntarily participated and signed the informed consent form.

### Study population

Retrospective analysis of general clinical and ultrasonographic data from 2599 children with CMT who received standardized treatment protocol at our hospital from 2004 to 2020. Additionally, electronic medical records of 2344 patients admitted due to CMT in 17 tertiary children’s hospitals of China’s Futang Research Center of Pediatric Development (FRCPD) from 2015 to 2019 were retrospectively analyzed.

### Inclusion and exclusion criteria

Inclusion criteria were: a. Abnormal SCM found by neck ultrasound, and the diagnosis was CMT. b. Typical clinical manifestations: history of head tilt or neck mass; head tilted to the affected side, mandible turned to the healthy side of the neck; head-face asymmetry and affected side slight; palpable SCM tension or thickening, neck Rotation, and lateral flexion are limited.

Exclusion criteria included a. Eye, vestibular, neurological, bone, neck inflammation or infection, and other clinical diseases as the cause of torticollis [1–3].

### Single-center study population and grouping

Children treated by our hospital’s CMT outpatient clinic from 2004 to 2020 were included. A total of 2599 children with CMT were finally enrolled.

#### Physiotherapy group

Children with CMT were divided according to SCM ultrasound characteristics into the local mass-type group (*n* = 1889), uniform thickening-type group (*n* = 684), and atrophy-type group (*n* = 26).

#### Physiotherapy combined with glucocorticoid treatment group

Furthermore, children with CMT were divided into the following groups according to whether they received local SCM injections of glucocorticoids: physiotherapy combined with glucocorticoid treatment group (*n* = 191) and physiotherapy without glucocorticoid treatment group (*n* = 2408).

#### Surgical treatment group

Children with CMT were divided into the following groups according to whether they eventually underwent surgical treatment: physiotherapy-ineffective conversion to surgery group (*n* = 231) and recovery after physiotherapy group (*n* = 2368).

### Multicenter study population

A total of 2344 patients who were surgically admitted due to CMT in 17 tertiary children’s hospitals of the FRCPD from 2015 to 2019 were included. We collected the electronic medical records of these patients and extracted data on sex, age, year of admission and discharge, and treatment cost during hospitalization from the first medical record pages according to the International Classification of Diseases (ICD) codes.

### Treatment programs

**Fig. 1:**
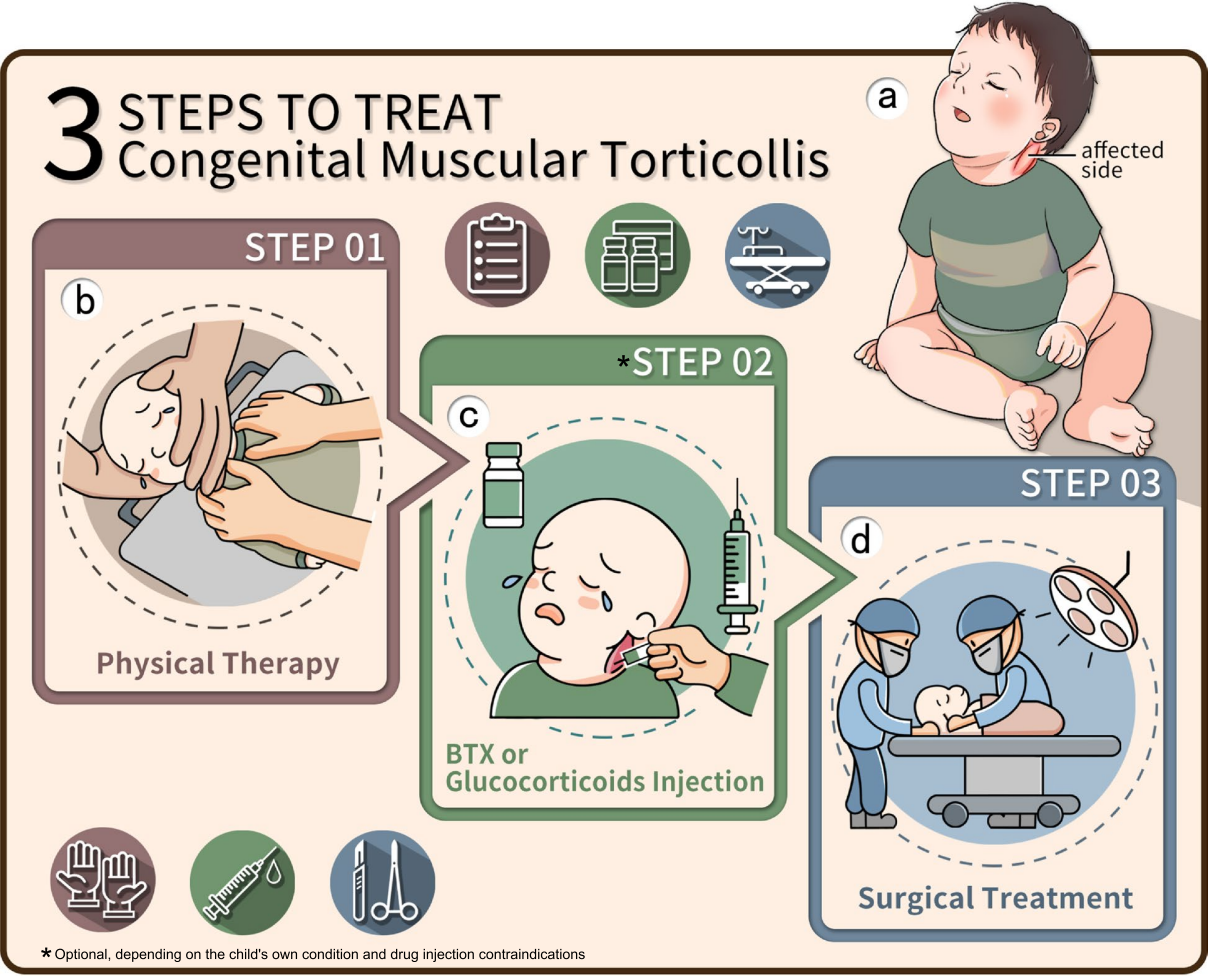
CMT standardized protocol of treatment. **a** Typical clinical manifestations of children with CMT. **b** Children diagnosed with CMT should receive standardized physiotherapy immediately. **c** Physiotherapy combined with glucocorticoid therapy. **d** Surgical treatment if conservative treatment fails

Training and experienced professionals treated children using standardized procedures [1] (Fig. 1).

#### Physiotherapy method [1]

The child removes the pillow and lies flat on his back on the treatment table. The child’s shoulders are flush with the edge of the treatment table. The physiotherapist stands next to the patient’s head. Hold the child’s head with both hands and keep the head in the air. The physiotherapist first evaluates the child's condition, including the size and hardness of the neck mass; the degree of activity of the neck. During treatment, the physiotherapist can first apply baby powder or non-irritating massage lubricant to the SCM on the affected side of the child to protect the skin. The physiotherapist’s hands are placed on the mandible’s healthy side and the occiput’s back. If the child has right-sided CMT, the physiotherapist’s right hand is placed on the back of the child’s pillow, and the left hand is placed on the child’s left mandible. During the operation, the physiotherapist’s left palm pushes the child’s lower jaw to the right shoulder with the help of strength, while the right hand cooperates with pulling. When the maximum rotation degree of the child’s neck is reached, the child's lower jaw can be supported and fixed for 2–3 s. During treatment, it is necessary to increase the intensity appropriately according to the severity of the child’s condition. During the physiotherapy process, the physiotherapist moderately pinched the local contracture of the child’s SCM with his fingers while observing the changes in the SCM. The treatment of left CMT is the same as that of the right. The entire physical therapy session takes about 10 min. The frequency of physiotherapy should be determined according to the child’s recovery, and it can be 2–5 times/per week. Humanistic care should be paid attention to during physiotherapy, and the movements are relatively gentle.

After about six courses (about three months) of the above-mentioned physiotherapy, the head and neck of children with CMT might be still skewed, and the neck mass might not become significantly smaller or softer. These children will be candidates to non-eco-guided local intramuscular injections of glucocorticoid, in association with physiotherapy. The operation process is as follows: the child takes the supine position, the neck is hyperextended, the head is turned to the uninjured side, and the SCM on the affected side is fully exposed. Routine disinfection, and laying towels, strictly abide by the principle of sterility. Because the SCM is located superficially, the deep layer is the carotid sheath, and the external jugular vein is on the outside. After the puncture, it should be confirmed that there is no blood return before injection to prevent direct injection into the vein. Inject glucocorticoid N mg/kg (total ≤ 20 mg) into the neck SCM mass. After the injection, the patient should be observed for 15 min before leaving the hospital without any adverse reactions. The physiotherapy was continued after a one-week interval, and the time interval between the two local injections of glucocorticoids was ≥ 2–3 months.

#### Surgical treatment

After continuous treatment with the above treatment protocol for more than six months, if the head and neck are still skewed, the neck mass is reduced but still hard, and the neck mobility has not improved significantly. Comprehensive conservative treatment is ineffective, and surgical treatment should be performed. In addition, if children with CMT have contraindications for local injection of glucocorticoids, such as allergic reactions, epilepsy, Cushing syndrome, or a history of surgery within three months, surgical treatment should be performed directly [18]. The operation process is as follows: the child takes the supine position, the neck is hyperextended, the head is turned to the uninjured side, and the SCM on the affected side is fully exposed. Routine disinfection and laying sterile sheets strictly abide by the principle of sterility. The surgeon made a transverse incision parallel to the clavicle 1 cm above the medial side of the clavicle on the affected side, about 3 cm long. The platysma is then divided, and the clavicular and sternal heads exposing the SCM are separated. The SCM’s clavicular and sternal head are then severed.

### Efficacy evaluation

When the child's neck is not skewed, the contracture mass of SCM on the affected side has completely dissipated, and the muscles are soft; the passive movement of the neck is not significantly restricted, and the difference in rotation between the healthy side and the affected side is within 5°. Patients need regular follow-up visits at three months, six months, and one year after treatment. The patient was considered to be cured without any clinical symptoms within one year [4–6].

### Statistical analysis

Data were organized and analyzed using SPSS software version 19.0. The data were assessed for normality using the Kolmogorov–Smirnov test. Quantitative data are expressed as mean ± standard deviation (x¯ ± s) and were analyzed using parametric tests, such as the t-test, or non-parametric tests, depending on the data distribution. Qualitative data are expressed as percentages (%) and were analyzed using the chi-square test or Fisher’s exact probability test, with α = 0.05 as the test level. *P* < 0.05 was considered to be indicative of statistically significant difference.

## Results

### Physiotherapy group

Based on the inclusion and exclusion criteria, 2599 children with CMT received the standardized protocol of treatment at the CMT specialist clinic of our hospital from 2004 to 2020 (Fig. 1).

**Fig. 2:**
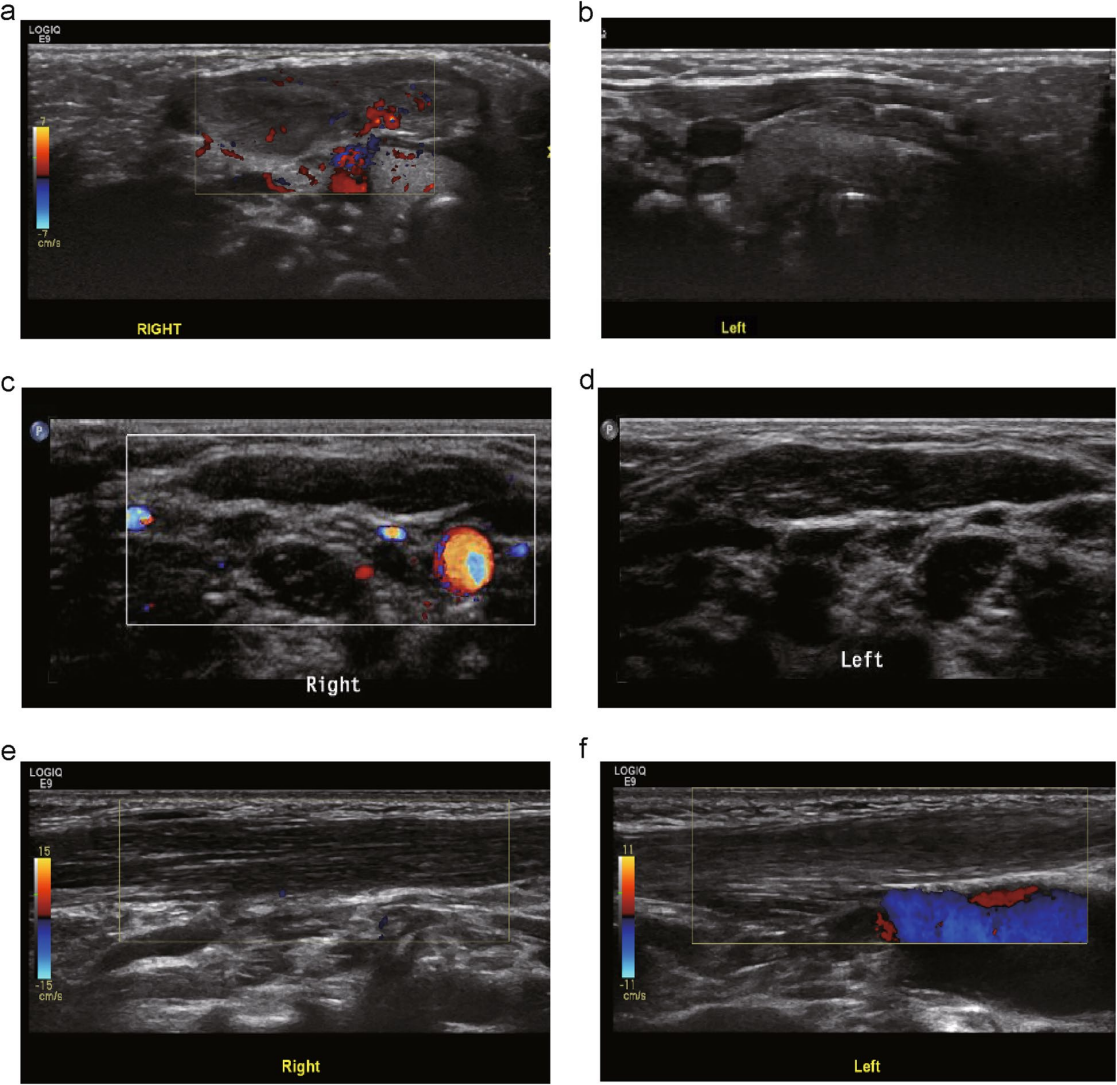
Imaging pictures of three types of ultrasound classification of CMT. **a** Local mass-type Ultrasound pictures of the affected side: the SCM of the affected side is thickened and nodular, with a size of about 3.4 × 3.0 × 1.1 cm. The echo is unevenly enhanced, the muscle texture is disordered, and the echo is unclear. **b** Local mass type Ultrasound pictures of the healthy side: the SCM echo of the healthy side shows no obvious abnormality, the muscle fiber texture is clear, and the thickness of the upper, middle and lower segments are 2.9 mm, 3.0 mm, and 3.0 mm, respectively. **c** Uniformly thickening type Ultrasound picture of the healthy side: no abnormality in the echo of the right SCM. The thickness of the middle section is 3.2 mm. **d** Uniformly thickening type Ultrasound picture of the affected side: the echo of the left SCM is enhanced and thickened, mainly in the lower part of the sternal bundle, and the thickness of the middle part is 4.0 mm. **e** Ultrasound image of the healthy side of atrophy type: the SCM echo of the healthy side (right) shows no abnormality, the muscle fiber texture is clear, and the thickness of the middle section is 7.3 mm. **f** Ultrasound image of the affected side of atrophy type: the affected side (left) has enhanced SCM echoes, mainly the sternal bundle, with a thickness of 5.2 mm in the middle

Our team defined three types of CMT according to the characteristics of SCM ultrasound examination—namely, local mass, uniform thickening, and atrophy (Fig. 2). With respect to the local mass type, the maximum thickness of the SCM was 32.62 ± 7.30 mm on the affected side and 3.82 ± 0.56 mm on the healthy side; the affected side-to-healthy side thickness ratio was 8.67 ± 2.29, with a thickness difference of 28.74 ± 7.33 mm between the affected and healthy sides. As for the uniform thickening type, the maximum thickness of the SCM was 7.22 ± 2.16 mm on the affected side and 4.27 ± 0.74 mm on the healthy side; the affected side-to-healthy side thickness ratio was 1.73 ± 0.57, with a thickness difference of 2.95 ± 2.16 mm between the affected and healthy sides. Regarding the atrophy type, the maximum thickness of the SCM was 3.23 ± 1.55 mm on the affected side and 4.73 ± 1.04 mm on the healthy side; the affected side-to-healthy side thickness ratio was 0.67 ± 0.27, with a thickness difference of -1.50 ± 1.21 mm between the affected and healthy sides (Table 1).

**Table 1:** Ultrasound data of three groups of ultrasound types in children with CMT

	N	Average	Standard Deviation	Min	Max	Percentbrant		
						25th percentile	median	75th percentile
**Local mass type**								
Maximum thickness of SCM on the affected side (mm)	1889	32.62	7.30	7.00	100.00	28.00	33.00	38.00
Maximum thickness of SCM on the healthy side (mm)	1889	3.82	0.56	1.30	6.70	3.50	3.80	4.10
Thickness ratio of the affected side to the healthy side	1889	8.67	2.29	1.78	30.00	7.18	8.57	10.00
Thickness difference between the affected and healthy sides (mm)	1889	28.74	7.33	3.30	95.60	24.10	28.90	33.50
**Uniformly thickening type**								
Maximum thickness of SCM on the affected side (mm)	684	7.22	2.16	3.60	16.00	5.70	6.80	8.20
Maximum thickness of SCM on the healthy side (mm)	684	4.27	0.74	2.40	7.40	3.80	4.20	4.70
Thickness ratio of the affected side to the healthy side	684	1.73	0.57	1.00	4.69	1.33	1.61	1.96
Thickness difference between the affected and healthy sides (mm)	684	2.95	2.16	0.00	11.90	1.40	2.55	4.00
**Atrophy type**								
Maximum thickness of SCM on the affected side (mm)	26	3.23	1.55	0.50	5.40	1.90	3.40	4.75
Maximum thickness of SCM on the healthy side (mm)	26	4.73	1.04	3.20	7.30	3.98	4.70	5.33
Thickness ratio of the affected side to the healthy side	26	0.67	0.27	0.14	0.98	0.46	0.77	0.91
Thickness difference between the affected and healthy sides (mm)	26	-1.50	1.21	-4.90	-0.10	-2.13	-1.50	-0.38

Age at first diagnosis was 69.21 ± 108.41 days in local mass type group, 216.85 ± 324.09 days in uniform thickening group, and 417.88 ± 739.05 days in atrophy- type group; while age at first physiotherapy use was 94.06 ± 206.49 days, 255.00 ± 430.62 days, 540.92 ± 1059.29 respectively. These results showed statistically significant differences (*P* < 0.05) (Table 2). Eventually, after standardized physiotherapy in our hospital, 141 children (7.5%) in the local mass-type group, 86 children (12.6%) in the uniform thickening-type group, and four children (15.4%) atrophy-type group underwent surgical treatment.

**Table 2:** Comparison of three groups of ultrasound typing treatments for children with CMT

	The local mass type subgroup (*N* = 1889)	The uniformly thickening type subgroup (*N* = 684)	The atrophy type subgroup (*N* = 26)	*P*-value
Age of first diagnosis	69.21 ± 108.41	216.85 ± 324.09	417.88 ± 739.05	< 0.05
Age of first use of physiotherapy	94.06 ± 206.49	255.00 ± 430.62	540.92 ± 1059.29	< 0.05
Total number of times of physiotherapy	40.63 ± 30.61	28.79 ± 26.49	40.81 ± 36.27	< 0.05
Frequency of physical therapy	0.25 ± 0.32	0.27 ± 0.40	0.20 ± 0.14	> 0.05
Total number of outpatient treatment days	40.63 ± 30.61	28.79 ± 26.49	40.81 ± 36.27	< 0.05
Outpatient cost	1625.23 ± 1224.19	1151.52 ± 1059.57	1632.31 ± 1450.89	< 0.05
Total hospitalization cost	6829.18 ± 2530.07	7606.58 ± 3225.18	8802.50 ± 4197.40	< 0.05
Operation	141 (7.50%)	86 (12.57%)	4 (15.38%)	-

In the physiotherapy group, the age at diagnosis was 112.44 ± 224.12 days, whereas the age at first physiotherapy use was 137.38 ± 312.11 days. The total number of times when physiotherapy was used was 35.68 ± 27.52, and the total number of days of outpatient treatment in our hospital was 35.66 ± 27.52. The outpatient cost was 1427.02 ± 1100.61 yuan, whereas the total hospitalization cost was 6818.25 ± 3497.11 yuan. For those with the uniform thickening type, the maximum thickness of the SCM was 7.14 ± 2.14 mm on the affected side. For the above-mentioned results, the differences were statistically significant at *P* < 0.05 (Table 3).

**Table 3:** Comparison of physiotherapy combined with glucocorticoid treatment group and physiotherapy without glucocorticoid treatment group

	Physiotherapy without glucocorticoid treatment group (*N* = 2408)	Physiotherapy combined with glucocorticoid treatment group (*N* = 191)	*P*-value
Age of first diagnosis	112.44 ± 224.12	115.87 ± 144.86	< 0.05
Age of first use of physiotherapy	137.38 ± 312.11	196.91 ± 344.26	< 0.05
Total number of times of physiotherapy	35.68 ± 27.52	60.69 ± 46.91	< 0.05
Frequency of physical therapy	0.26 ± 0.35	0.24 ± 0.21	> 0.05
Total number of outpatient treatment days	35.66 ± 27.52	60.69 ± 46.91	< 0.05
Outpatient cost	1427.02 ± 1100.61	2427.64 ± 1876.18	< 0.05
Total hospitalization cost	6818.25 ± 3497.11	7833.76 ± 1545.44	< 0.05
Local mass type-the size of the SCM mass on the affected side (mm^3^)	10255.61 ± 5596.66	10977.31 ± 5529.71	> 0.05
Local mass type-the maximum thickness of SCM on the affected side (mm)	32.59 ± 7.40	33.11 ± 6.02	> 0.05
Uniformly thickening type-the maximum thickness of SCM on the affected side (mm)	7.14 ± 2.14	8.30 ± 2.17	< 0.05
Atrophy type-the maximum thickness of SCM on the affected side (mm)	3.28 ± 1.56	2.10 ± 0.00	> 0.05
Maximum thickness of SCM on the healthy side (mm)	3.95 ± 0.66	4.06 ± 0.64	< 0.05
Thickness ratio of the affected side to the healthy side	6.63 ± 3.75	6.77 ± 3.46	> 0.05
Thickness difference between the affected and healthy sides (mm)	21.11 ± 13.44	22.09 ± 12.48	> 0.05
Operation	155 (6.43%)	76 (39.79%)	-

### Physiotherapy combined with glucocorticoid treatment group

Out of 2599 children with CMT who underwent standardized physiotherapy in our hospital, 191 received combined glucocorticoid therapy. In the physiotherapy combined with glucocorticoid treatment subgroup, the age at diagnosis was 115.87 ± 144.86 days, whereas the age at first physiotherapy use was 196.91 ± 344.26 days. Furthermore, the total number of times when physiotherapy was used was 60.69 ± 46.91, and the total number of days of outpatient treatment in our hospital was 60.69 ± 46.91. The outpatient cost was 2427.64 ± 1876.18 yuan, whereas the total hospitalization cost was 7833.76 ± 1545.44 yuan. For those with the uniform thickening type, the maximum thickness of the SCM was 8.30 ± 2.17 mm on the affected side, and 76 patients (39.8%) underwent surgery. For the above-mentioned results, the differences were statistically significant at *P* < 0.05 (Table 3).

### Surgical treatment group

Out of 2599 children with CMT who received standardized physiotherapy in our hospital, 231 were converted to surgery because physiotherapy was ineffective and 2368 were cured after physiotherapy. In the physiotherapy-ineffective group, the age at diagnosis was 160.88 ± 189.76 days, whereas the age at first physiotherapy use was 162.69 ± 190.36 days. The frequency of physiotherapy use was 0.21 ± 0.20. The size of the SCM mass on the affected side was 12,891.15 ± 6912.75 mm in the local mass type. The maximum thickness of the SCM on the affected side was 34.86 ± 6.96 mm in the partial mass type and 7.95 ± 1.97 mm in the uniform thickening type. The maximum thickness of the SCM on the healthy side was 4.20 ± 0.71 mm.

In the recovery after physiotherapy group, the age at diagnosis was 107.40 ± 221.03 days, whereas the age at first physiotherapy use was 140.02 ± 326.06 days. The frequency of physiotherapy use was 0.26 ± 0.35. The size of the SCM mass on the affected side was 10,104.11 ± 5423.68 mm in the local mass type. The maximum thickness of the SCM on the affected side was 32.44 ± 7.30 mm in the partial mass type and 7.12 ± 2.17 mm in the uniform thickening type. The maximum thickness of the SCM on the healthy side was 3.94 ± 0.65 mm. For the above-mentioned results, the differences were statistically significant at *P* < 0.05 (Table 4).

**Table 4:** Comparison of the subgroup of physiotherapy ineffective conversion to surgery and the recovery subgroup after physiotherapy

	the subgroup of physiotherapy ineffective conversion to surgery (*N* = 231)	the recovery subgroup after physiotherapy (*N* = 2368)	*P*-value
Age of first diagnosis	160.88 ± 189.76	107.40 ± 221.03	< 0.05
Age of first use of physiotherapy	162.69 ± 190.36	140.02 ± 326.06	< 0.05
Total number of times of physiotherapy	37.81 ± 31.78	37.49 ± 29.92	> 0.05
Frequency of physical therapy	0.21 ± 0.20	0.26 ± 0.35	< 0.05
Total number of outpatient treatment days	37.79 ± 31.85	37.47 ± 29.92	> 0.05
Outpatient cost	1512.38 ± 1271.08	1499.43 ± 1196.79	> 0.05
Local mass type-the size of the SCM mass on the affected side (mm^3^)	12891.15 ± 6912.75	10104.11 ± 5423.68	< 0.05
Local mass type-the maximum thickness of SCM on the affected side (mm)	34.86 ± 6.96	32.44 ± 7.30	< 0.05
Uniformly thickening type-the maximum thickness of SCM on the affected side (mm)	7.95 ± 1.97	7.12 ± 2.17	< 0.05
Atrophy type-the maximum thickness of SCM on the affected side (mm)	3.03 ± 1.59	3.27 ± 1.57	> 0.05
Maximum thickness of SCM on the healthy side (mm)	4.20 ± 0.71	3.94 ± 0.65	< 0.05
Thickness ratio of the affected side to the healthy side	6.00 ± 3.87	6.70 ± 3.71	< 0.05
Thickness difference between the affected and healthy sides (mm)	19.65 ± 14.69	21.33 ± 13.22	< 0.05

### Multicenter study population

**Fig. 3:**
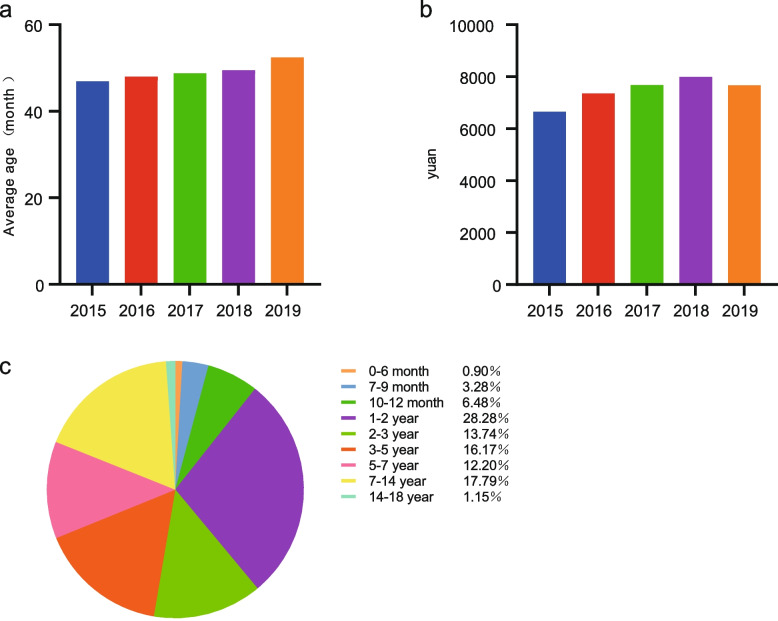
Information on the age of surgery and treatment costs of patients admitted for CMT surgery in 17 tertiary children’s hospitals in FRCPD from 2015 to 2019. **a** Histogram shows the average age of surgery from 2015 to 2019. **b** Histogram shows the average cost of surgical treatment from 2015 to 2019. **c** Patients are grouped by age. Circle chart shows the proportion of children in different age groups

A total of 2344 children with CMT who were admitted to 17 tertiary children’s hospitals of the FRCPD from 2015 to 2019 were included. We collected the first medical record pages according to the ICD codes, extracted the corresponding medical record information, and obtained data on sex, age, year of admission and discharge, time of visit, and various expenses during hospitalization. Among children, 1376 and 968 were male and female, respectively. The average age at surgery was 46.94 months in 2015, 47.98 months in 2016, 48.76 months in 2017, 49.49 months in 2018, and 52.41 months in 2019. The 5-year average age at surgery was 50.00 months, and the overall trend was increasing year by year (Fig. 3A). The average hospitalization cost was 6654.28 yuan in 2015, 7352.68 yuan in 2016, 7679.68 yuan in 2017, 7997.25 yuan in 2018, and 7674.62 yuan in 2019. The 5-year average hospitalization cost was 7646.77 yuan (Fig. 3B). We grouped the 2344 children with CMT according to age. Overall, 21 children (0.9%) were 0–6 months of age; 77 children (3.3%) were 7–9 months of age; 152 children (6.5%) were 10–12 months of age; 663 children (28.3%) were 1–2 years of age; 322 children (13.7%) were 2–3 years of age; 379 children (16.2%) were 3–5 years of age; 286 children (12.2%) were 5–7 years of age; 417 individuals (17.8%) were 7–14 years of age; and 27 individuals (1.2%) were 14–18 years of age (Fig. 3C).

## Discussion

CMT is a common congenital musculoskeletal disease that affects infants and young children. Summarizing the experiences for treating CMT will aid in improving the management of CMT and reduce the teratogenicity rate. The present study retrospectively analyzed 2599 children with CMT who received the standardized treatment plan at our hospital from 2004 to 2020; subsequently, these children were grouped according to ultrasound-related characteristics and treatment outcomes. Based on the characteristics of SCM ultrasound examination in CMT, this study defined three types of CMT—namely, local mass, uniform thickening, and atrophy. Additionally, the epidemiological and economic burden data of patients admitted for CMT surgery in 17 tertiary children’s hospitals of the FRCPD from 2015 to 2019 were retrospectively analyzed in this study.

A typical feature of CMT is the presence of a hard, irregularly shaped mass in the neck at approximately 14 days after birth in most children. Such mass is primarily distributed along the SCM [1–3]. The natural history of patients with CMT can be roughly divided into three situations: (a) The SCM mass completely disappears and heals, or the SCM mass disappears but remains with fascicular contracture and head deviation. (b) In some children, the entire SCM exhibits cord-like thickening and hardening, even though the SCM has never had a mass. (c) The proliferation of both fibrous and adipose tissues in CMT leads to muscle atrophy caused by calpain and the ubiquitin–proteasome system [11]; that is, the SCM of patients with CMT will have muscle atrophy.

**Fig. 4:**
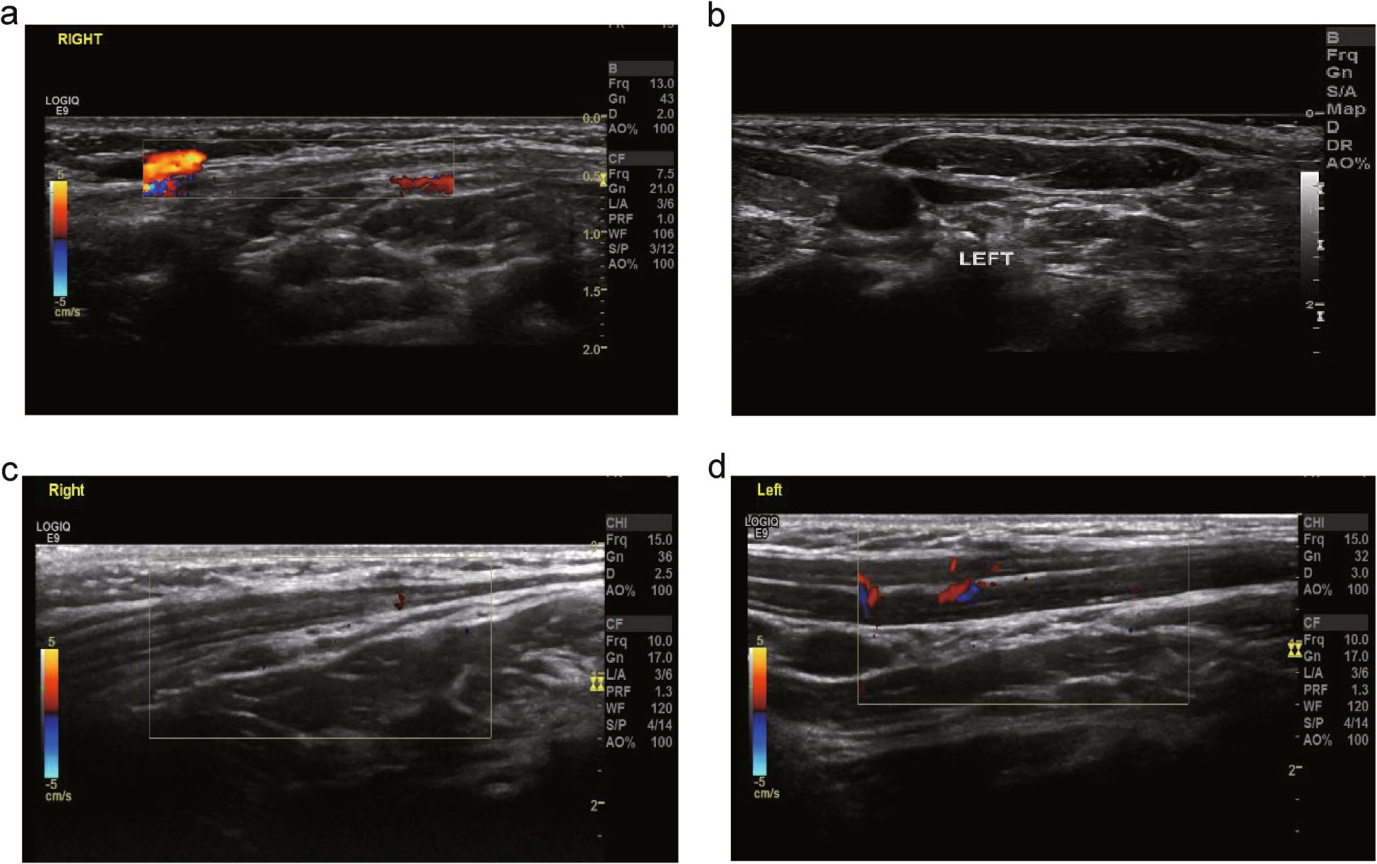
SCM ultrasound results of short-term observation (more than one year) in children with atrophy type. **a** At the first visit: diffuse thinning of the SCM on the affected side, mainly the sternal bundle, poor internal echo, clear muscle texture, and the thinnest part in the middle and the lower segment is about 1.5 mm. **b** At the first visit: the echo of the SCM on the healthy side is well-proportioned, and the thickness of the upper, middle and lower segments are 4.6 mm, 4.7 mm, and 4.7 mm, respectively. **c** One year later: the SCM of the affected side is diffusely thinned, mainly the sternal tract, the echo is uneven, and the muscle texture is still evident. The thickness of the upper, middle, and lower segments are 4.3 mm, 2.8 mm, and 1.7 mm, respectively. **d** Reexamination after one year: the SCM echo of the healthy side is uniform, and the thickness of the upper, middle, and lower segments are 4.5 mm, 4.6 mm, and 4.6 mm, respectively

The diagnosis of CMT usually relies on the clinical presentation and physical examination. Ultrasonography can clearly show the location, size, shape, and internal echo of SCM lesions in children with CMT [2, 7]. Our team defined three types of CMT according to the characteristics of SCM ultrasound examination: local mass, uniform thickening, and atrophy. The ultrasound characteristics of the local mass type included an apparent nodular mass in the SCM on the affected side, uneven echo enhancement, muscle texture disorder, and unclear echo [4, 19]. The ultrasound characteristics of the uniform thickening type included cord-like thickening of the entire SCM on the affected side, enhanced echo, less uniformity, less clear muscle fiber texture, and no obvious space-occupying echoes [4, 19]. The ultrasound characteristics of the atrophic type included a considerably thinner SCM on the affected side than on the healthy side, as well as enhanced but less uniform echo. Additionally, the muscle fiber texture echo was less clear (Fig. 2). Notably, we found for the first time that some children with CMT had the atrophy type. After standard treatment and follow-up for 1 year, patients with atrophy-type CMT showed no significant differences in bilateral SCM thickness (Fig. 4).

It has been observed that children with CMT have the potential for self-resolution during the early stages of the condition [5, 9, 10, 13]. Nevertheless, early treatment methods remain controversial. However, the sooner children with CMT receive standardized treatment, the better their prognosis will become [20]. Physiotherapy remains the first choice and most applied treatment [5, 7, 9]. From 2004 to 2020, 2599 children with CMT were treated at our hospital with physiotherapy combined with local injection of SCM drugs and SCM release surgery in the standardized protocol of treatment (Fig. 1). After receiving standardized physiotherapy in our hospital, 141 children in the local mass type group (7.5%), 86 children in the uniform thickening-type group (12.6%), and four patients in the atrophy-type group (15.4%) underwent surgical treatment; the overall conservative treatment success rate was as high as 91.1%. Additionally, the age at first diagnosis and age at first physiotherapy use were generally earlier in the local mass-type group. Only 141(7.5%) of children underwent surgical treatment. These results suggest that early diagnosis and treatment of children with CMT are crucial for their prognosis.

After three months of physiotherapy, if the head and neck of children with CMT may still be skewed, there is no apparent change in the neck mass [18, 21]. In that case, these children with CMT will be combined with glucocorticoid local intramuscular injection therapy. Out of 2599 children with CMT who received standardized physiotherapy in this study, 191 received physiotherapy combined with glucocorticoid therapy, and 76 (39.8%) underwent surgery. Based on the treatment results, physiotherapy combined with glucocorticoid therapy could accelerate the regression of SCM masses, promote muscle softening, and reduce fibrous contractures [18]. However, the dose and interval of administration should be strictly controlled, and the occurrence of complications should be considered. As children constantly grow and develop, long-term follow-up is necessary, even if the short-term curative effect is satisfactory. We also found that children in the physiotherapy without glucocorticoid treatment group generally had an earlier age at first diagnosis and physiotherapy use. Eventually, only 155 children (6.43%) underwent surgical treatment in the physiotherapy without glucocorticoid treatment group. These results suggest that early diagnosis and treatment of children with CMT are crucial for their prognosis (Table 3).

Cheng et al. [13] reported that after at least 6 months of physiotherapy, if children still had head and neck tilt, passive neck rotation, and lateral flexion > 15° and that SCM tension or SCM mass size and hardness did not subside, surgical treatment is needed timely. Yu et al. [22] quantitatively and qualitatively assessed the craniofacial deformities in patients with uncorrected CMT. Their results indicated that skull and skull base deformities appeared earlier, whereas facial deformities appeared later and tended to aggravate with age. Surgical treatment should be performed before the child turns 1 year of age to prevent or reverse head deformities [23]. Some scholars believe that premature surgical treatment, especially before 1 year of age, may cause problems in postoperative wound management and is more likely to produce hematoma and increase infection; therefore, they believe that the best time for surgery in children with CMT is 1–4 years of age [5, 24]. Shim et al. [24] believed that the essential factor for determining the optimal timing for surgery in children is not age but compliance with the postoperative rehabilitation plan and suggested that the age at surgery in children can be appropriately postponed. The indications for CMT surgery and the selection of the best timing for surgery are inconsistent. Further studies with more robust evidence are required to comprehensively treat children with CMT. We found that the age at first diagnosis and the age at first physiotherapy use in children who recovered after physiotherapy were usually earlier, suggesting that early diagnosis and treatment of children with CMT are crucial to their prognosis. We believe that facial asymmetry and head deformities can improve after professional surgical treatment in children of any age [25]. If children with CMT have indications for surgery, operation should be performed despite the age. If the contracted SCM is earlier released, craniofacial deformities may not present [1]. Additionally, when deciding whether to perform surgery, it is necessary to comprehensively consider age, whether one has received regular physiotherapy, severity of head and face deformity, head tilt, angle of head and neck rotation, and lateral flexion limitation to guide the treatment of CMT.

This study included 2344 children who underwent surgery for CMT at 17 tertiary children’s hospitals of the FRCPD between 2015 and 2019. The mean age at surgery in CMT children was 50.00 months. Among them, 663 children with CMT aged 1–2 years accounted for the largest proportion (28.3%), followed by 417 children aged 7–14 years (17.8%), indicating that there are still more children with CMT receiving surgical treatment later. The above results indicate that we should strengthen missionary education and emphasize standardized treatment.

The study presents some limitations. The retrospective design may introduce information distortion risk and retrospective bias. In addition, some confounding variables could not be controlled during the data collection. This study only has data from a single center in our hospital regarding CMT ultrasound classification. Our team is preparing a prospective multi-center study with much detailed data.

In conclusion, early diagnosis and treatment are essential to improve the conservative treatment success rate and achieve good prognosis in children with CMT. Our standardized treatment protocol primarily involves regular physiotherapy, with glucocorticoid injections and SCM release surgery added when necessary. This program has a high conservative treatment success rate and may facilitate the achievement of better prognosis and reduced teratogenicity rate.

## Data Availability

The datasets generated or analyzed during the study are available from the corresponding author on reasonable request.
